# Classifying central serous chorioretinopathy subtypes with a deep neural network using optical coherence tomography images: a cross-sectional study

**DOI:** 10.1038/s41598-021-04424-z

**Published:** 2022-01-10

**Authors:** Jeewoo Yoon, Jinyoung Han, Junseo Ko, Seong Choi, Ji In Park, Joon Seo Hwang, Jeong Mo Han, Kyuhwan Jang, Joonhong Sohn, Kyu Hyung Park, Daniel Duck-Jin Hwang

**Affiliations:** 1 Department of Applied Artificial Intelligence, Sungkyunkwan University, Seoul, Korea; 2 RAON DATA, Seoul, Korea; 3 Department of Medicine, Kangwon National University Hospital, Kangwon National University School of Medicine, Chuncheon, Gangwon-do Korea; 4 Seoul Plus Eye Clinic, Seoul, Korea; 5 Kong Eye Center, Seoul, Korea; 6 Department of Ophthalmology, Hangil Eye Hospital, 35 Bupyeong-daero, Bupyeong-gu, Incheon, Korea; 7 Department of Ophthalmology, Seoul National University Bundang Hospital, Seongnam, Korea; 8 Department of Ophthalmology, Catholic Kwandong University College of Medicine, Incheon, Korea; 9 LUX MIND, Incheon, Korea

**Keywords:** Medical imaging, Eye diseases

## Abstract

Central serous chorioretinopathy (CSC) is the fourth most common retinopathy and can reduce quality of life. CSC is assessed using optical coherence tomography (OCT), but deep learning systems have not been used to classify CSC subtypes. This study aimed to build a deep learning system model to distinguish CSC subtypes using a convolutional neural network (CNN). We enrolled 435 patients with CSC from a single tertiary center between January 2015 and January 2020. Data from spectral domain OCT (SD-OCT) images of the patients were analyzed using a deep CNN. Five-fold cross-validation was employed to evaluate the model’s ability to discriminate acute, non-resolving, inactive, and chronic atrophic CSC. We compared the performances of the proposed model, Resnet-50, Inception-V3, and eight ophthalmologists. Overall, 3209 SD-OCT images were included. The proposed model showed an average cross-validation accuracy of 70.0% (95% confidence interval [CI], 0.676–0.718) and the highest test accuracy was 73.5%. Additional evaluation in an independent set of 104 patients demonstrated the reliable performance of the proposed model (accuracy: 76.8%). Our model could classify CSC subtypes with high accuracy. Thus, automated deep learning systems could be useful in the classification and management of CSC.

## Introduction

Central serous chorioretinopathy (CSC) is the fourth most common retinopathy following age-related macular degeneration (AMD), diabetic retinopathy, and branch retinal vein occlusion^1^. The incidence of CSC is 9.9 per 100,000 men and 1.7 per 100,000 women^1–3^. Persisting subretinal fluid (SRF) damages the outer layer of the retina, thus causing permanent vision loss, which degrades the quality of life^4–6^. In addition, CSC is reportedly associated with polypoidal choroidal vasculopathy^7–10^, a subtype of neovascular AMD, and its importance is growing.

CSC is typically classified as acute or chronic, according to the chronicity of the disease and the pattern of retinal pigment epithelium (RPE) change. However, it is difficult to identify the aforementioned categories using one imaging test^5^. Therefore, CSC is traditionally diagnosed using multimodal imaging modalities, such as fundus photography (FP), fluorescein angiography (FA), indocyanine green angiography (ICGA), optical coherence tomography (OCT), and fundus autofluorescence (AF)^5,11^. Among these modalities, FA and ICGA are essential for accurately diagnosing CSC. However, they have limitations owing to their invasive and time-consuming nature. In contrast, OCT is a non-invasive, rapid, and accurate test, and generates highly reproducible results^11–13^. It is now considered a gold standard imaging modality for the follow-up of patients with CSC^5^.

Recently, a deep learning approach was applied to differentiate CSC from normal eyes and classify acute and chronic CSC using OCT^14^, which demonstrates the predictive power of the deep learning model similar to retina specialists. This study goes one step further; instead of just differentiating acute or chronic CSC, we propose a deep convolutional neural network (CNN) that can classify the subtypes of CSC using OCT image alone. In clinical practice, various CSC subtypes have been reported that are not easily differentiated by retina experts^5,15^. Hence, a detailed diagnosis and classification would enable better patient understanding of the prognosis, according to the CSC subtype. Moreover, this would facilitate the selection of more appropriate treatment policies and management^11,14^.

There is neither a universally accepted classification system for CSC nor a consensus on features that constitute chronic CSC^15,16^. In addition, no research has distinguished the detailed CSC subtypes using deep learning models. Herein, based on Daruch's report^15^, we classified CSC into four subtypes, namely acute, non-resolving, inactive, and chronic atrophic CSC. We then proposed a CNN that could classify CSC into the aforementioned subtypes. We aimed to compare the performance of the proposed model with that of ophthalmologists and applied gradient weighted class activation mapping (Grad-CAM)^17^ with the aim of determining the characteristic features used by the model in the classification process.

## Results

We included 3209 images of 435 patients with CSC. The mean age was 52.76 ± 9.65 years. Table 1 summarizes the baseline characteristics of the enrolled patients.

**Table 1:** Baseline characteristics of patients who underwent macular optical coherence tomography.

	CSC^a^				
	Acute	Non-resolving	Chronic	Inactive	Total
Images, *n*	929	887	764	629	3209
Patients, *n*	109	107	109	110	435
Age, years, mean (SD^b^)	49.31 (8.40)	52.91 (9.56)	58.29 (8.76)	50.59 (9.44)	52.76 (9.65)
**Gender, *n*** (%)					
Male	95 (87.16)	82 (76.64)	98 (89.91)	76 (69.10)	351 (80.69)
Female	14 (12.84)	25 (23.36)	11 (10.09)	34 (30.90)	84 (19.31)
**Eye, *n* (%)**					
Right	45 (41.28)	53 (49.53)	58 (53.21)	59 (53.64)	215 (49.43)
Left	64 (58.72)	54 (50.47)	51 (46.79)	51 (46.36)	220 (50.57)

^a^*CSC* central serous chorioretinopathy.

^b^*SD* standard deviation.

### Model performance

**Figure 1:**
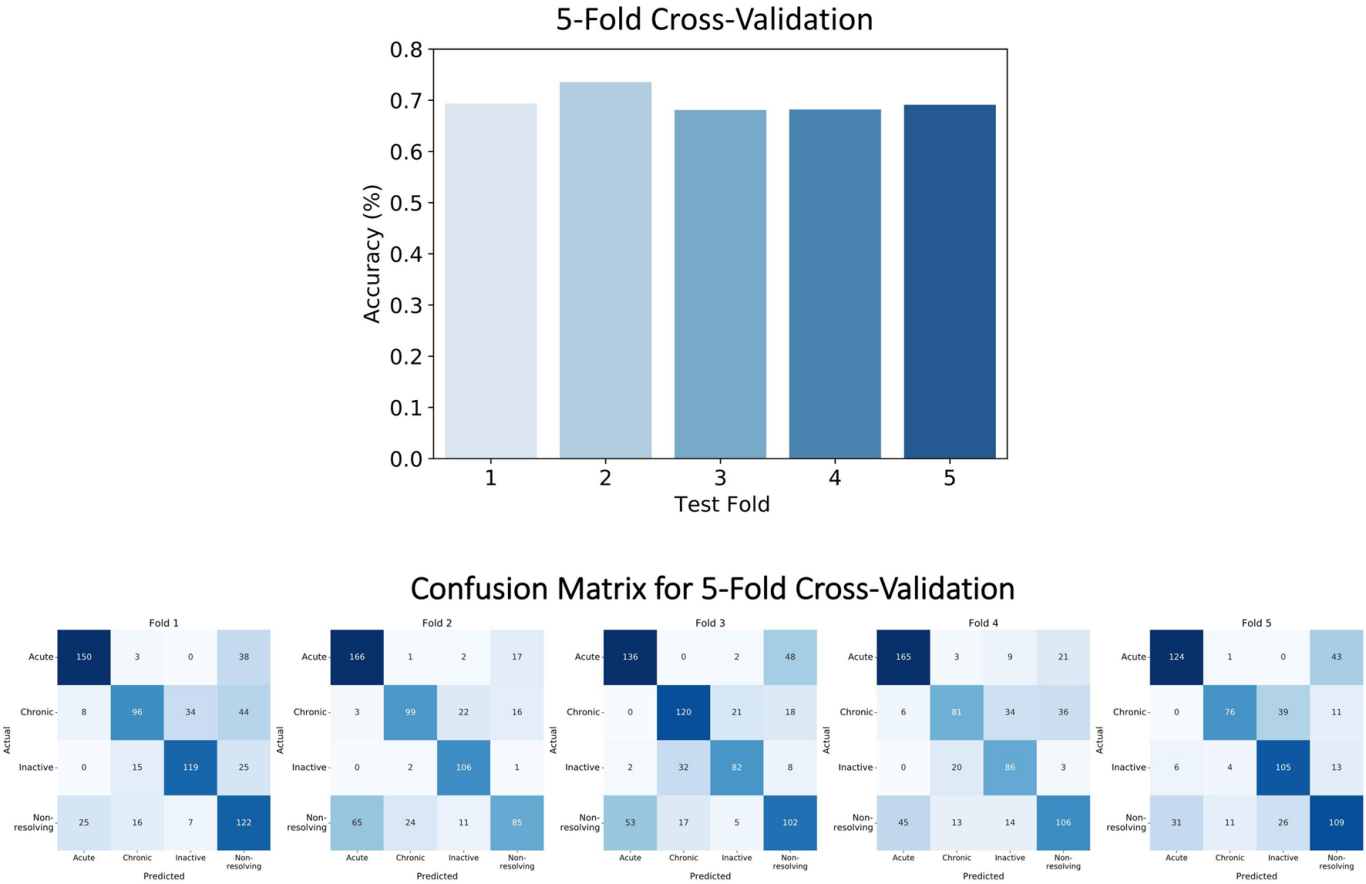
Performance comparison of models on spectral domain-optical coherence tomography images. Test accuracies of each fold during five-fold cross-validation. Our proposed model achieved the highest accuracy of 73.5% when using the second fold as the test set. An illustration of the confusion matrix of classification results in five-fold cross-validation. While the x-axis denotes the predicted class, the y-axis denotes the ground truth. Therefore, diagonal values from the top left to bottom right denote the correct predictions made by our proposed model. Our proposed model displays robust performance on every fold.

The proposed model, based on VGG-16, for classifying CSC subtypes demonstrated an average cross-validation accuracy of 70.0% (95% confidence interval [CI], 0.676–0.718), which was higher than that of Resnet-50 (68.6%; 95% CI, 0.643–0.730) and Inception-V3 (68.2%; 95% CI, 0.653–0.712). Moreover, we applied the transfer learning method to Resnet-50 and Inception-V3. Our model achieved the highest test accuracy of 73.5% on providing the second fold to the test set (Fig. 1). Figure 1 also depicts the confusion matrix for the five-fold cross-validation results. Our proposed model revealed more false categorizations while classifying acute and non-resolving subtypes than while classifying other subtypes. Moreover, we collected 665 OCT images from 104 patients, which included 161, 197, 134, and 173 OCT images for acute, non-resolving, chronic, and inactive cases, respectively, to evaluate the model’s performance on an additional independent dataset. The proposed model achieved a high accuracy on the independent dataset (76.8%).

### Performance comparison with ophthalmologists

**Figure 2:**
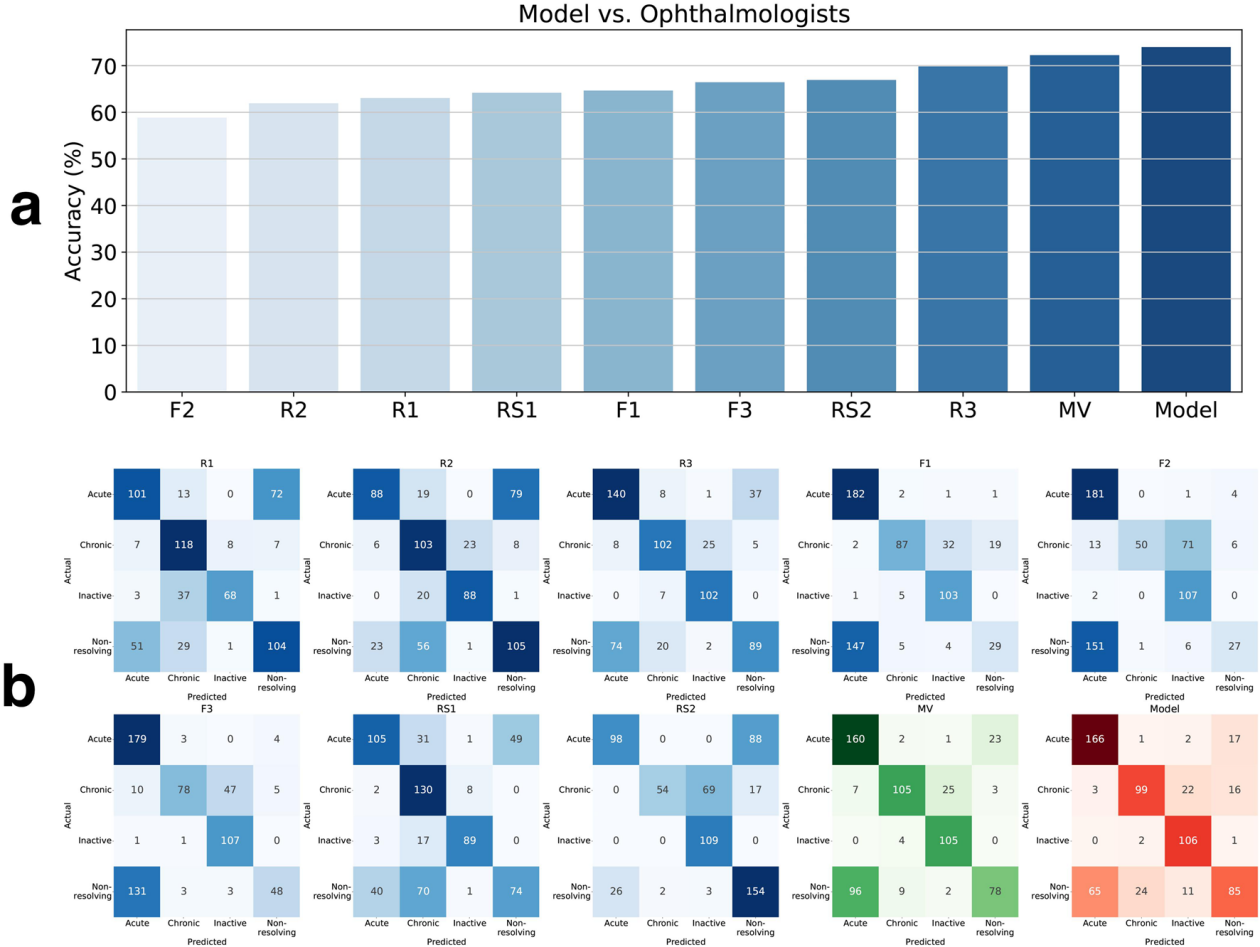
Performance comparison between the proposed model and the ophthalmologists. (**a**) Accuracy comparison between our proposed model and the ophthalmologists in distinguishing the four different central serous chorioretinopathy subtypes. While our proposed model has achieved a high accuracy of 73.5%, the ophthalmologists (including majority vote [MV]) achieved accuracies of 58.9–72.3%. (**b**) Confusion matrix comparison between our model and the ophthalmologists in distinguishing the four different central serous chorioretinopathy subtypes. All subjects, i.e., the eight ophthalmologists, majority vote, and the proposed model generated more false cases in distinguishing acute and non-resolving subtypes than other retinal disease pairs. R1, R2, and R3 denote ophthalmology residents with < 1, 3, and 4 years of experience, respectively. F1, F2, and F3 denote retina fellows with < 1, 2, and 2 years of experience, respectively. RS1 and RS2 refer to retina specialists (RS) with > 10 years of experience. MV refers to the majority vote results obtained by eight ophthalmologists. *F* retina fellow, *R* ophthalmology resident.

Figure 2 represents the performance of the ophthalmologists and the proposed model for classifying the four different CSC subtypes. The classification accuracy of eight ophthalmologists for diagnosing the four CSC subtypes ranged from 58.9 to 72.3% (including the majority vote). In contrast, our model displayed an accuracy of 73.5%. Therefore, the proposed model demonstrated better performance than that of the ophthalmologists in classifying the four CSC subtypes based on OCT images. In particular, among the 102 OCT images that the two retina specialists failed to correctly diagnose into the four CSC subtypes, our model correctly categorized 65 OCT images (64%). Of the 223 OCT images that one retina expert analyzed correctly, the proposed model accurately classified 140 OCT images (63%). Similar to the proposed model, most ophthalmologists failed to correctly distinguish the acute and non-resolving subtypes (Fig. 2b). However, among the 93 OCT images that the two retina specialists failed to categorize into acute and non-resolving images, our model could correctly categorize 61 images (66%). Among the 125 OCT images that one retina expert classified correctly, our model correctly classified 76 images (61%). That is, our proposed model could capture the key features of retinal diseases from the OCT images, thus displaying great utility in supporting ophthalmologists in their diagnoses. However, among the 153 OCT images classified by the two retina specialists into acute and non-resolving CSC, the model failed to correctly classify 39 images (25.4%, Table 2). The Kappa coefficients between the first and second retina specialists, the model and first retina specialist, and the model and second retina specialist were 0.47, 0.46, and 0.51, respectively, all of which demonstrated moderate agreement (*P* < 0.001) in diagnosing the four CSC subtypes.

**Table 2:** Case comparison between the proposed model and human experts.

						
Model	Non-resolving	Non-resolving	Non-resolving	Acute	Chronic	Acute
R1^a^	Acute	Acute	Non-resolving	Non-resolving	Acute	Non-resolving
R2^a^	Acute	Acute	Non-resolving	Acute	Non-resolving	Non-resolving
R3^a^	Acute	Non-resolving	Acute	Acute	Non-resolving	Acute
F1^b^	Acute	Acute	Acute	Acute	Acute	Acute
F2^b^	Acute	Acute	Acute	Acute	Acute	Acute
F3^b^	Acute	Acute	Acute	Acute	Acute	Acute
RS1^c^	Acute	Acute	Acute	Non-resolving	Non-resolving	Non-resolving
RS2^c^	Acute	Acute	Acute	Non-resolving	Non-resolving	Non-resolving
GT^d^	Acute	Acute	Acute	Non-resolving	Non-resolving	Non-resolving

The six images denote the false detections by our model while classifying images into central serous chorioretinopathy subtypes.

^a^R1, R2, and R3 denote ophthalmology residents with < 1, 3, and 4 years of experience, respectively.

^b^F1, F2, and F3 denote retina fellows with < 1, 2, and 2 years of experience, respectively.

^c^RS1 and RS2 refer to retina specialists with > 10 years of experience.

^d^GT denotes the ground truth.

### Grad-CAM visualization

**Figure 3:**
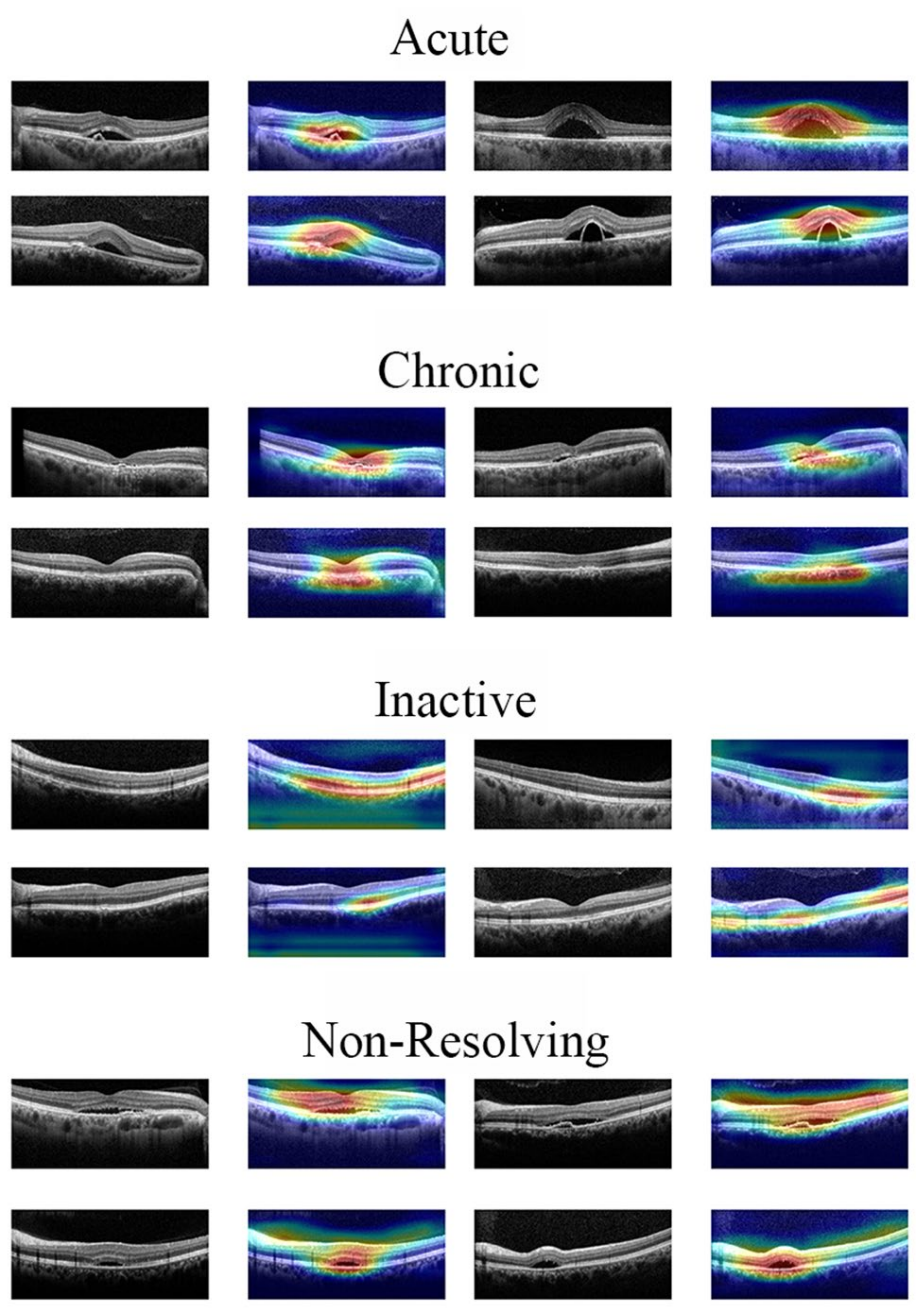
Heat maps for the classification models by gradient weighted class activation mapping (Grad-CAM). Grad-CAM was able to identify the pathologic regions of central serous chorioretinopathy (CSC) on the optical coherence tomography (OCT) images, presented as a heat map.

Figure 3 depicts the representative heat maps produced by the Grad-CAMs. The highlighted regions were those where the retina specialists usually considered diagnosing CSC subtypes^11,14^. The heat maps revealed that our proposed model used an approach similar to that used by retina specialists in assessing the CSC images.

## Discussion

We aimed to build a deep learning model and investigate its performance for classifying CSC subtypes, without a segmentation algorithm. Our model could effectively distinguish the CSC subtypes and its performance was either comparable to or better than that of experienced retina doctors.

Our model displayed an overall good performance across all subtypes, particularly in detecting acute, inactive, and chronic CSC (Fig. 2b). Despite several reports on CSC treatment, there is no consensus on its classification and optimal treatment^5,15,16,18,19^. This gap in the knowledge necessitates a large-scale prospective randomized controlled trial in the future. This is because CSC has a relatively high proportion of spontaneous improvement or resolution, which can be mistaken for a therapeutic effect in retrospective studies^5^. Traditionally, in acute or inactive CSC, clinicians recommend observation without immediate treatment as the initial management^16^. That is, in acute CSC, immediate interventions, such as focal laser treatment, intravitreal anti-vascular endothelial growth factor injections, or photodynamic therapy, are unnecessary. However, it is necessary to monitor the reduction of SRF via follow-up^5,15,16^. In case of inactive CSC, treatment may not be required. Nonetheless, there lies a possibility of CSC recurrence with SRF, thereby necessitating regular follow-up. Non-resolving CSC is likely to become chronic with extensive atrophic changes, particularly during irreversible damage to the photoreceptor with persistent SRF. In such cases, active intervention supposedly prevents permanent vision impairment. Moreover, in the case of chronic CSC, treatment is generally considered in the presence of SRF^16,20,21^. However, severe irreversible atrophic change in the photoreceptor may hinder the recovery of visual function. Thus, treatment may not be meaningful.

In this study, we added the inactive type to the classification system, which was classified as a case with a history of a previous CSC episode and RPE irregularity, but no definite atrophic change^15,22^. The proposed model distinguished chronic atrophic and inactive CSC using a single OCT image, demonstrating better performance than that of ophthalmologists (Fig. 2b). Inactive CSC does not require specific treatment but requires regular follow-up. Furthermore, the visual prognosis is good. Therefore, despite the lack of SRF in inactive CSC, it is necessary to differentiate the subtype from chronic CSC with no SRF. The latter is characterized by atrophic damage to the photoreceptors and RPE, resulting in deterioration of vision quality.

It was most difficult to distinguish between acute and non-resolving CSC using only OCT images. Particularly, the two retina experts misclassified 52% and 32% of the actual acute or non-resolving CSCs, compared to our model’s incorrect answer rate of 32%. The major reason was that the distinction between acute and non-resolving CSC by Daruich et al.^15^ was not based on OCT but was arbitrarily based on patient symptoms over a period of 4 months. Second, only one OCT image was used without multimodal imaging information, possibly leading to low diagnostic performance. Nevertheless, our model could correctly identify 61 images (66%) of the 93 images that both retina experts classified incorrectly. Of the 125 images on which the two retina experts disagreed (i.e., only one retina expert answered correctly), 76 could be correctly identified by our model (61%). Acute and non-resolving CSC are arbitrarily divided based on a specific timeline of 4 months. However, they differ in the duration of SRF. Thus, an OCT-based biomarker may exist but has not yet been reported. Hence, our model could learn the latent pattern and display better performance than ophthalmologists. That is, the proposed deep learning model could assist retina experts in subtype classification (i.e., acute vs. non-resolving CSC) that requires skilled experience.

In certain macular diseases, such as CSC, Grad-CAM^17^ could be an adjunctive tool for detecting OCT biomarkers while determining the characteristic features of the macula^14^. Using Grad-CAM, we were able to identify and specify the parts of an image that affect each of the CSC subtype probability scores. The heat map of the regions activated by the model could identify and quantify the differences, highlighting the crucial areas’ classification process. First, in acute CSC, our model did not consider the significance of an increase in choroidal thickness but instead considered the retina important. The shape change in the inner retinal layer deformed by SRF rather than the SRF itself was considered an important criterion for subtype classification. In the first image of acute CSC in Fig. 3, RPE detachment (RPED) was primarily observed. Interestingly, our model did not highlight the base of the RPED, rather highlighted its upper boundary and the retina located above. This supported our assumption that greater inner retina change above the photoreceptor layer was considered an important criterion for identifying acute CSC. Second, our model principally examined the outer layer of the retina and the choroid for classifying chronic CSC. Compared to other subtypes, the choroid appears to be an important clue in the identification of chronic CSC. Among its several layers, the choriocapillaris and Sattler’s layer, which correspond to the inner choroid, appear to be primarily emphasized. Third, in inactive CSC, the model focused on the RPE and the photoreceptors of the outer layer of the retina. In contrast, the inner layer of the retina and the choroid appeared unimportant. Inactive CSC is defined as a case comprising RPE alterations without SRF. This necessitates the assessment of changes in the RPE for diagnosing inactive CSC. As demonstrated in the second inactive CSC case in Fig. 3, the upper portion of the photoreceptor layer, such as the inner nuclear layer and outer plexiform layer, was simultaneously highlighted. That is, the RPE and photoreceptors are predominantly considered critically. However, clinicians consider the inner layers of the photoreceptor while classifying inactive CSC. The Grad-CAM visualization suggested that our model had similar standards to those of ophthalmologists. In addition, in non-resolving CSC, our model seemed to observe both the retina and the choroid. In particular, the inner layers of the retina (i.e., from the internal limiting membrane to the outer nuclear layer) were more widely emphasized than the area highlighted in acute CSC. In general, acute CSC accompanies only changes in the choroid or the outer retinal layer, including the RPE, while considering its pathogenesis^5,15^. From the Grad-CAM results, we could infer the possibility of the accompanying subtle abnormal changes in the inner retinal layer with prolonged SRF duration, as it became the non-resolving type. Therefore, it is necessary to determine if structural change in the retinal inner layer follows with time when SRF persists for several months in non-resolving CSC.

**Figure 4:**
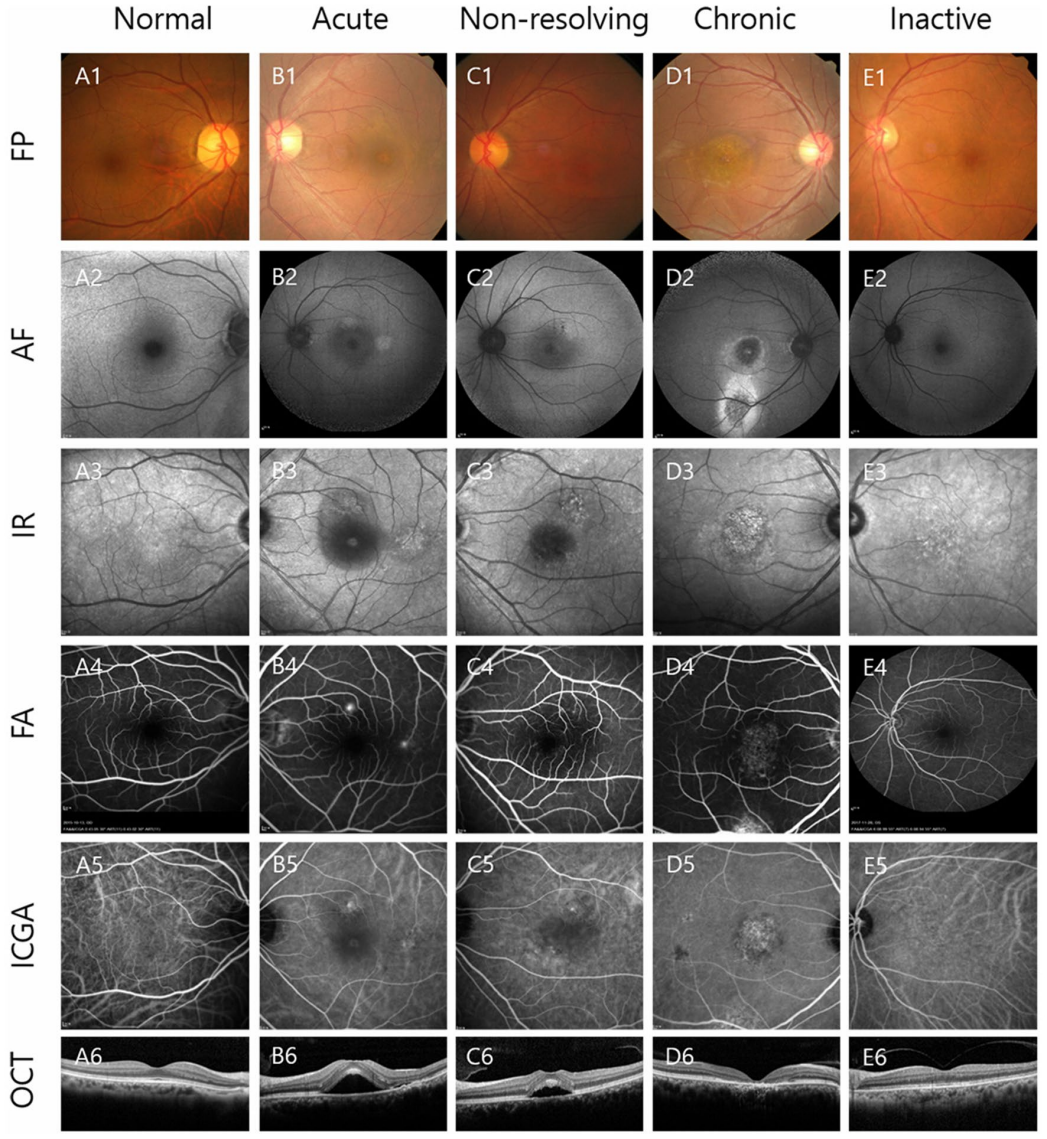
Representative cases of CSC according to the subtypes: Normal (A), Acute (B), Non-resolving (C), Chronic atrophic (D), and Inactive CSC (E). In acute CSC (B), IR (B3) showed decreased reflectance on foveal center due to SRF. FA (B4) shows leakage of dye in ink blot pattern and OCT shows (B6) increased subfoveal choroidal thickness and presence of subretinal fluid. In non-resolving CSC (C), FA (C4) shows minimal leakage of dye and OCT (C6) shows subretinal fluid with RPE undulation. In chronic atrophic CSC (D), AF (D2) shows gravitational track of RPE atrophy and OCT (D6) shows disrupted outer retinal layer with atrophic changes of photoreceptor layer. In inactive CSC (E), AF (E2) and OCT (E6) shows RPE irregularity without atrophy. IR (E3) image shows multiple granular pattern. *FP* Color fundus photography, *AF* Fundus autofluorescence, *IR* Infrared, *FA* Fluorescein angiography, *ICGA* Indocyanine green angiography, *OCT* Optical coherence tomography.

Our study had several limitations. First, we included only OCT images from patients who met our inclusion criteria and trained the deep neural network with these images. We excluded patients with other concomitant macular diseases. External validation should be performed with images from different OCT manufacturers in future studies. All images were acquired from a single OCT device located at an academic center. However, the dataset was sufficient to demonstrate the feasibility of our model to distinguish the CSC subtypes. Second, we investigated the model’s performance using the information obtained from one OCT image per case. Thus, information from multiple OCT images was not considered. In clinical practice, ophthalmologists typically make a final diagnosis by observing several OCT images along with clinical information, such as symptom duration. Therefore, diagnosing CSC by combining multiple images with diverse clinical information would be more accurate than considering one image. We compared the performance of the model and ophthalmologists with one cropped OCT image, which may not be identical to the actual clinical environment. This necessitates extending our model to the actual clinical setting. In addition, for classifying acute and non-resolving CSC, the classification performance would likely be improved by using not only the OCT B scan image, but also the infrared (IR) reflectance image, which is automatically provided by the Spectralis OCT device. The IR reflectance decreased (B3) because of SRF in acute CSC (Fig. 4). The foveal granular pattern (E3) in inactive CSC indicated a long-term change in RPE melanin distribution, following a CSC episode. IR or near-IR images are other non-invasive imaging modalities based on autofluorescence, emanating predominantly from RPE and choroid melanin^23,24^. Training the deep learning model with multimodal imaging information, such as OCT, FP, IR, FA, and ICGA, will more clearly help distinguish between an acute type with relatively fresh SRF and a non-resolving type that has persisted for longer. Hence, a deep learning model that comprehensively learns from several images per case or additional multimodal images (i.e., IR, FAG, or ICGA images) simultaneously can achieve a higher level of performance than the current model. Third, we only focused on classifying CSC subtypes in this cross-sectional analysis. The model could be extended to predict the treatment response or vision prognosis according to CSC subtypes using the longitudinal image dataset of patients with CSC.

We aimed to develop a deep neural network model to classify four CSC subtypes. To diagnose these subtypes, we cast the problem as a multinomial classification issue. To solve the multinomial classification problem, we utilized the soft-max activation function^25^, a generalization of the sigmoid function^25^ used in binary classification, at the end of the proposed model. The proposed multinomial classification model displayed higher accuracy than the ophthalmologists, thereby demonstrating great utility in clinical support for the precise diagnosis of retinal diseases.

In summary, we developed a deep CNN model that could distinguish CSC subtypes with > 70% accuracy. Such an efficient automatic classification model could play a subsidiary role in supporting retina experts in distinguishing and managing various CSC subtypes. Our findings provide a basis for developing accurate OCT-based models for diagnosing and managing CSC and other macular diseases.

## Methods

### Ethics

This study was conducted in accordance with the tenets of the Declaration of Helsinki. The Ethics Committee of Hangil Eye Hospital approved the research protocol and its implementation (Hangil IRB—20,007). The committee waived the requirement for informed consent, considering the retrospective observational study design.

### Data collection and labeling

We analyzed the records of patients who visited the Hangil Eye Hospital between January 2015 and January 2020. Independent retina specialists diagnosed all CSC cases using fundus examinations and FP, AF, IR, FA, ICGA, and OCT images. A confocal scanning laser ophthalmoscope (Heidelberg Retina Angiograph, HRA; Heidelberg Engineering, Heidelberg, Germany) was used to perform simultaneous FA and ICGA in all cases.

We used the spectral domain (SD)-OCT (Heidelberg Spectralis, Heidelberg Engineering, Heidelberg, Germany) images of 435 patients with CSC. We selected one eye per patient, and included images from a single visit per patient. Moreover, we excluded data that revealed the presence of other potentially conflicting retinal pathologies, such as AMD, polypoidal choroidal vasculopathy, pachychoroid neovasculopathy, and pachychoroid pigment epitheliopathy. We randomly selected one to five non-centered image cuts from 25 volume scan image cuts for each OCT volume and five centered image cuts displaying the typical CSC pattern.

We used the modified version of Daruich et al.’s CSC classification scheme^15^. Based on the RPE or photoreceptor status and the duration of symptoms, patients were classified into one of the following CSC subtypes: (1) acute CSC with SRF lasting ≤ 4 months; (2) non-resolving CSC with SRF persisting > 4 months; (3) chronic atrophic CSC with definite RPE and photoreceptor atrophy, with or without SRF; and (4) inactive CSC without SRF, but with signs of a previous CSC episode with RPE irregularity (Table 3 and Fig. 4).

**Table 3:** Modified central serous chorioretinopathy classification according to the subretinal fluid, retinal pigment epithelium, and photoreceptor status^15,22^.

CSC^a^ subtype	SRF^b^	SRF duration	RPE^c^ status
Acute	Yes	≤ 4 months	Normal RPE or RPE alterations without atrophy
Non-resolving	Yes	> 4 months	Normal RPE or RPE alterations without atrophy
Chronic atrophic	Yes or No	NA^d^	Widespread (or focal) RPE and photoreceptor atrophy with or without gravitational tracks
Inactive	No	NA	Signs of previous CSC episode with RPE alterations without atrophy

^a^*CSC* central serous chorioretinopathy.

^b^*SRF* subretinal fluid.

^c^*RPE* retinal pigment epithelium.

^d^*NA* not available.

Two retina specialists (DDH and JMH) examined the medical records and performed categorization. All images obtained using OCT, FA, and ICGA multimodal imaging methods were included in this study. In cases of disagreement, a third retina specialist (JSH) evaluated the discrepancy and discussed the case with other specialists. Following discussion, all discrepancies were resolved by consensus.

### Data preprocessing

We prepared 3,209 SD-OCT images as an input for the deep neural network by cropping each 596 × 1264-sized SD-OCT image into a 340 × 746-sized RGB image. We subsequently down-sampled the cropped 340 × 746 image to a 224 × 224 RGB image for input into the deep neural network, which only accepted the fixed-sized images. Interestingly, 224 × 224 RGB is widely used for popular image classification models, such as VGG-16^26^ and Resnet-50^27^. To avoid overfitting, we performed data augmentation for building a robust model on a variety of input images. In particular, the data augmentation process included the following steps: (i) random horizontal image flips, (ii) random width/height image shift in the range of [-1.0, 1.0] pixels, and (iii) random rotations up to 15° of the image. We performed the data augmentation process only in the training phase.

### Model architecture

**Figure 5:**
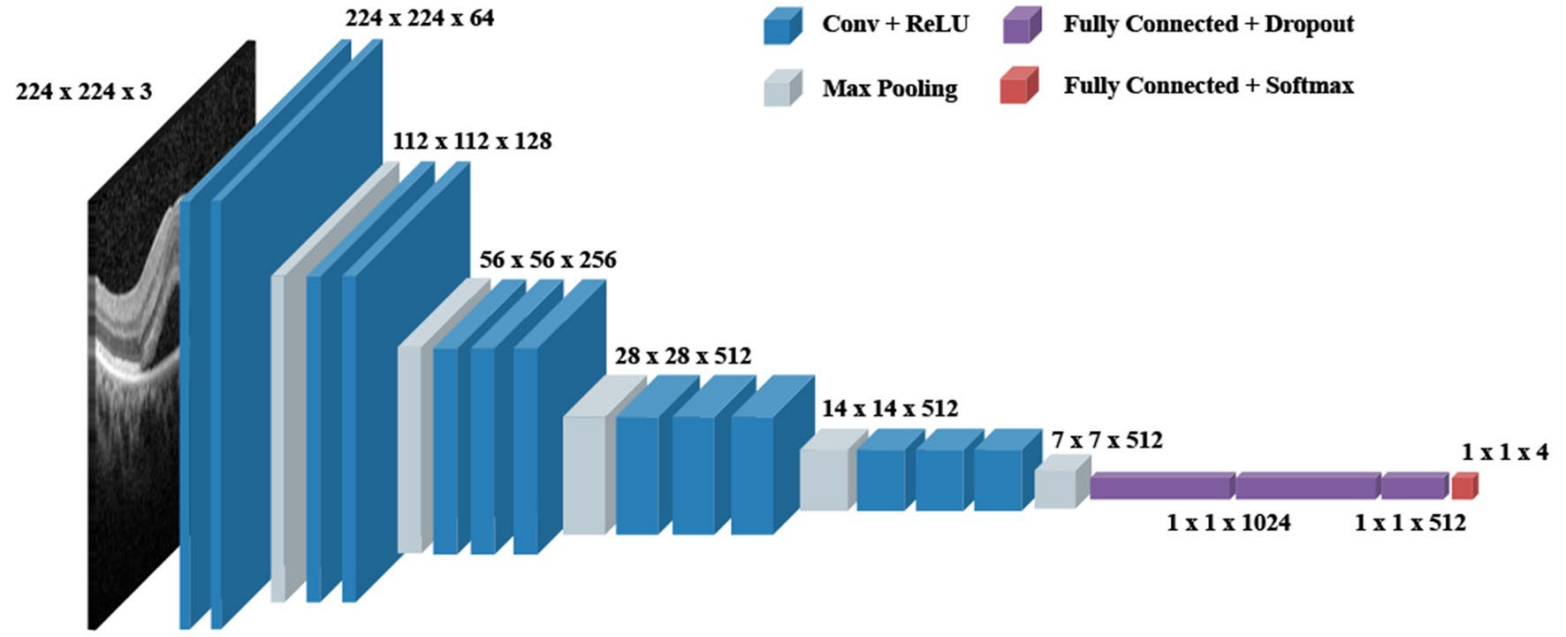
An illustration of the proposed model based on VGG-16 architecture. Our proposed model consists of an input layer, 16 convolutional neural network layers with ReLU activation functions, five max-pooling layers, four FC layers with dropouts, and soft-max. The final FC layer with a soft-max activation function has been used to predict one of the four subtypes, i.e., acute, chronic, in-active, and non-resolving central serous chorioretinopathy.

To classify a specific OCT image into four different retinal diseases, we built a deep learning model, based on the well-known CNN architecture, VGG-16^26^. Despite other well-known CNN architectures, including Resnet-50^27^ and Inception-V3^28^, we selected VGG-16 considering its better performance. The proposed model consisted of 13 CNN layers, followed by a rectified linear unit (ReLU) activation function, five max-pooling layers, and four completely connected layers with dropouts and soft-max activation (Fig. 5). While the dropout can help to avoid overfitting, a fully connected layer is a traditional multi-layered perceptron^29^. We used the final output layer with a soft-max activation function^25^ at the end of the proposed model, to predict one of the four subtypes. Herein, the soft-max activation function converted a raw prediction vector in the range of [− infinity, + infinity] (e.g., [2.0, 1.0, 0.5, 0.2]) from the logits layer to a vector of multiple categorical probabilities in the range of [0, 1] (e.g., [0.6, 0.2, 0.1, 0.1]). Interestingly, 0.6, 0.2, 0.1, and 0.1 denoted the probability that the given OCT image belonged to the first, second, third, and fourth categories, respectively. The model subsequently compared the soft-max outputs with one-hot encoded labels to calculate the cross-entropy loss^25^. If the index of the highest value of the soft-max outputs did not match the correct (ground-truth) label index, the proposed model obtained a penalty and updated its weights.

Furthermore, we applied the transfer learning method to avoid overfitting, and to train our model faster^30^. Specifically, we initialized 13 CNN layers with pre-trained weights, which were components of VGG-16 and acquired from the large-scale dataset ImageNet^31^. Consequently, we froze the CNN layers during training to reduce the computation time. The proposed deep neural network comprised 27,267,588 trainable parameters.

### Grad-CAM visualization

We applied Grad-CAM to visualize the pathological region of an OCT image^17^. Grad-CAM highlighted the important regions in the OCT image when our proposed model classified the target label (acute, chronic, inactive, and non-resolving). We determined the activated regions using the gradients for the feature maps of the CNN layer. Moreover, we created a heat map to highlight the area of the image used by the model for classification.

### Experiment setup

To train and evaluate the proposed model, we performed five-fold cross-validation. We split our dataset into five different folds, and subsequently trained the model with four folds. The model was then tested with the remaining fold. We conducted five classification rounds, each of which used a different test fold, and calculated the average value of the performance metrics for the five rounds. We split the datasets by patients. That is, not a single patient existed across different folds. To compare the model performance with that of other well-known CNN architectures, including Resnet-50^27^ and Inception-V3^28^, we conducted five cross-validations on these models. All models (including the proposed model) were trained with a batch size of 64, epochs of 30, and Adam optimization^32^ (learning rate: 0.0001).

To evaluate the proposed model from a clinical perspective, we selected the classification round that demonstrated the best performance in the five-fold cross-validation and provided the test fold data of the selected round to eight ophthalmologists, including three ophthalmology residents, three retina fellows, and two retina specialists, each with > 10 years of clinical experience at an academic ophthalmology center. They reviewed a test fold comprising 186, 140, 109, and 185 OCT images for acute, chronic, inactive, and non-resolving cases, respectively. To obtain the collective opinion of multiple ophthalmologists for each case, we reported on the eight ophthalmologists’ majority consensus. For example, if six ophthalmologists identified an OCT image as non-resolving and the other two ophthalmologists classified it as inactive, the image would be classified as non-resolving.

### Statistical analyses

We used Cohen’s Kappa coefficients to rate the agreement level between the two retina experts. We calculated this statistic using Scikit-learn, a well-known Python library.

## Data Availability

The data are not available for public access because of patient privacy concerns, but are available from the corresponding author on reasonable request.
